# Hyperthermic intravesical chemotherapy with gemcitabine versus Bacillus Calmette–Guerin in intermediate- and high-risk non-muscle-invasive bladder cancer

**DOI:** 10.3389/fonc.2025.1715762

**Published:** 2026-01-05

**Authors:** Bing Yan, Wenya Hu, Yanran Li, Tao Feng, Lei Zhu, Haifeng Xu, Shubo Chen, Yuhua Qiao

**Affiliations:** Department of Urology, Xingtai People’s Hospital, Xingtai, Hebei, China

**Keywords:** bladder cancer, hyperthermic intravesical chemotherapy, gemcitabine, Bacillus Calmette–Guerin, hyperthermia

## Abstract

**Background:**

The recurrence rate remains high in patients with intermediate- and high-risk non-muscle-invasive bladder cancer (NMIBC), and some patients may experience disease progression despite curative trans-urethral resection and adjuvant therapy.

**Objective:**

To compare the outcomes of intermediate- and high-risk NMIBC patients treated with Bacillus Calmette–Guerin (BCG) versus hyperthermic intravesical chemotherapy (HIVEC) with gemcitabine (GEM).

**Methods:**

A retrospective analysis of our single-institutional, prospectively collected database from July 2018 to February 2020 was performed. Patients with intermediate- and high-risk NMIBC and treated with HIVEC/GEM or BCG were selected. The two adjuvant therapies were compared in terms of recurrence-free survival (RFS), progress-free survival (PFS), and cancer-specific survival (CSS) at 24 months. Adverse events were also assessed and compared between groups.

**Results:**

A total of 85 patients (38 in the HIVEC/GEM group and 47 in the BCG group) were included in the study. Patients’ characteristics were comparable between groups. There were no statistically significant differences in RFS (78.0% for HIVEC/GEM versus 75.0% for BCG, *p* = 0.592), PFS (91.7% for HIVEC/GEM versus 94.6% for BCG, *p* = 0.670), and CSS at 24 months. AEs were less common with HIVEC/GEM treatment than BCG (47.37% (18/38) versus 70.20% (33/47), *p* = 0.033). Most AEs were mild with CTCAE grade 1-2 (42.1% (16/38) for HIVEC/GEM and 63.8% (30/47) for BCG, *p* = 0.046).

**Conclusions:**

Among patients with intermediate- and high-risk NMIBC, hyperthermic intravesical chemotherapy combined with gemcitabine, compared with BCG treatment, significantly reduced the adverse events rate without compromising oncological survival outcomes. The primary limitations of this study are its small sample size and retrospective nature.

## Introduction

Bladder cancer is the tenth most commonly diagnosed cancer worldwide (1). Among them, about 75% of newly diagnosed patients were with non-muscle-invasive diseases. Long-term prognosis of survival in non-muscle-invasive bladder cancer (NMIBC) patients is usually good, but recurrence and progress rates within 5 years can be as high as 50% and 20% in some patients, respectively, depending on risk stratification (2). For intermediate- and high-risk NMIBC patients, adjuvant therapy after transurethral resection (TURB) is necessary. Bacillus Calmette–Guerin (BCG) has been the standard care during the past 2 decades, but various new strategies have also been under investigated for further improving the prognosis (1). One of the promising new strategies is hyperthermic intravesical chemotherapy (HIVEC), which remarkably enhanced the function of traditional intravesical chemotherapy and provided comparable or even better oncological outcomes towards BCG in some clinical research (3–6). However, most of the current proofs were based on HIVEC using mitomycin C (MMC), the optimal reagent combined with hyperthermia and/or the regime is still under investigation.

Gemcitabine (GEM) is a novel intravesical instillation agent. Efficacy of GEM versus BCG has been investigated in several randomized trials (7–9). There were no significant differences in the recurrence and progression rates between GEM and BCG in intermediate-risk patients, but GEM had a higher recurrence rate than BCG in high-risk patients (9). Meanwhile, GEM is an ideal drug for HIVEC due to its good thermal stability, which is supported by several studies reporting promising results for the combination of GEM and hyperthermia (10–12). In this study, we report the results of HIVEC using GEM compared to BCG in patients with intermediate- and high-risk NMIBC.

## Materials and methods

### Patient selection

We retrospectively reviewed our single-institutional, prospectively maintained database that consecutively collected bladder cancer patients treated with transurethral resection followed by HIVEC/GEM or BCG instillation between July 2018 and February 2020. Inclusion criteria were patients with a diagnosis of intermediate- and high-risk NMIBC as defined by the 2016 version of EAU guidelines. Exclusion criteria were (i) carcinoma *in situ*, which has been proven to be insensitive to intravesical chemotherapy; (ii) histopathologies other than urothelial carcinomas; (iii) relapsed disease, with less than 24 months of disease-free periods since the last treatment; (iv) a short (< 24 months) or irregular follow-up. This study was conducted in accordance with the principles of the Declaration of Helsinki (as revised in 2024). In view of the retrospective nature of the study, informed consent to participate was waived by the local Ethics Committee of Xingtai People’s Hospital. The reporting of this study conforms to STROBE guidelines (13).

### Treatment schedules

Before adjuvant intravesical therapy, patients first experienced complete transurethral resection of all visible tumoral lesions. A re-TURB will be given 4 weeks after the initial resection for patients with T1/high-grade Ta tumors or absence of detrusor muscle in the pathology specimen.

Both two intravesical therapies were elaborately consulted with the patients; options were taken according to the patient’s decision. In the HIVEC/GEM procedure, HIVEC was administered using the BR-TRG-I urinary bladder hyperthermia treatment system (Bright Medical Technology Co., Ltd., Guangzhou, China) combined with 1000 mg GEM diluted in 50 mL distilled water. The total dwell time was 60 min at a target temperature of 43 ± 0.5°C. Patients received 8 induction sessions (weekly), followed by 10 maintenance sessions (monthly) for the remainder of year 1. In the BCG procedure, the intravesical instillation was given according to a 1-year schedule, which included 6 weekly and 3 fortnightly induction sessions, followed by 10 monthly maintenance sessions. Patients retained BCG in the bladder for 2 hours. Both adjuvant therapies in both groups started 2–4 weeks postoperatively.

Patients’ follow-up was at least 24 months after the last TURB. Cystoscopy and urine cytology were performed quarterly for the first two years, followed by semiannually for three years, and annually thereafter. Abdominal and pelvic computerized tomography urography was performed annually or if clinically indicated. TURB was performed if a suspicious lesion was detected during cystoscopy or imaging scan.

### Outcomes

The primary outcome of the study was the comparison of recurrence-free survival (RFS) at 24-month following TURB and two different adjuvant therapies. The secondary outcomes included Progression-free survival (PFS) and cancer-specific survival (CSS). Adverse events (AEs) were also routinely recorded and evaluated according to the Common Terminology Criteria for Adverse Events (CTCAE) version 5.0) (14).

### Statistical analysis

Descriptive statistics were used to test possible differences in baseline and pathologic characters between the HIVEC/GEM and BCG groups. Continuous and categorical variables were compared with Student’s t test, Mann–Whitney tests, or chi-squared tests, respectively. Survival analysis was performed using the Kaplan–Meier method and compared using the Wilcoxon test. The hazard ratio (HR) and its 95% confidence interval (CI) were calculated. Statistical analysis was performed using SPSS Statistics v.26.0 (IBM Corp., Armonk, NY, USA) software.

All tests were two-sided, with statistical significance defined as p ≤ 0.05.

## Results

A total of 85 patients with intermediate- and high-risk papillary NMIBC (38 in the HIVEC/GEM group and 47 in the BCG group) were identified. Baseline patient characteristics are shown in **Table 1**. No significant differences were identified between HIVEC/GEM and BCG in terms of median age, gender, and tumor characteristics (**Table 1**). High-risk NMIBC was 63.2% in the HIVEC/GEM group versus 48.9% in the BCG group. All patients had a minimum follow-up time of 24 months. The median follow-up times of the HIVEC/GEM and BCG groups were 30 (IQR: 26-37) and 32 (IQR: 31-36) months, respectively (*p* = 0.120).

**Table 1:** Patients’ demographic and tumor characteristics.

Characteristics	HIVEC/GEM (n=38)	BCG (n=47)	*p* value
Age, years, median(IQR)	58(51,67)	64(55,68)	0.263
Males, n(%)	35(92.1)	40(85.1)	0.511
Stage, n(%)			0.128
Ta	18(47.4)	30(63.8)	
T1	20(52.6)	17(36.2)	
Tumor grade, n(%)			0.530
Low grade	22(57.9)	24(51.1)	
High grade	16(42.1)	23(48.9)	
Number of tumors, n(%)			0.976
Single	29(76.3)	36(76.6)	
Multiple	9(23.7)	11(23.4)	
Tumor size, n(%)			0.577
< 3cm	22(57.9)	30(63.8)	
≥ 3cm	16(42.1)	17(36.2)	
Risk group, n(%)			0.190
Intermediate	14(36.8)	24(51.1)	
High	24(63.2)	23(48.9)	
Follow up, months, median(IQR)	30(26,37)	32(31,36)	0.120

HIVEC, hyperthermic intravesical chemotherapy; GEM, gemcitabine; BCG, Bacillus Calmette–Guerin; IQR, interquartile range.

**Figure 1:**
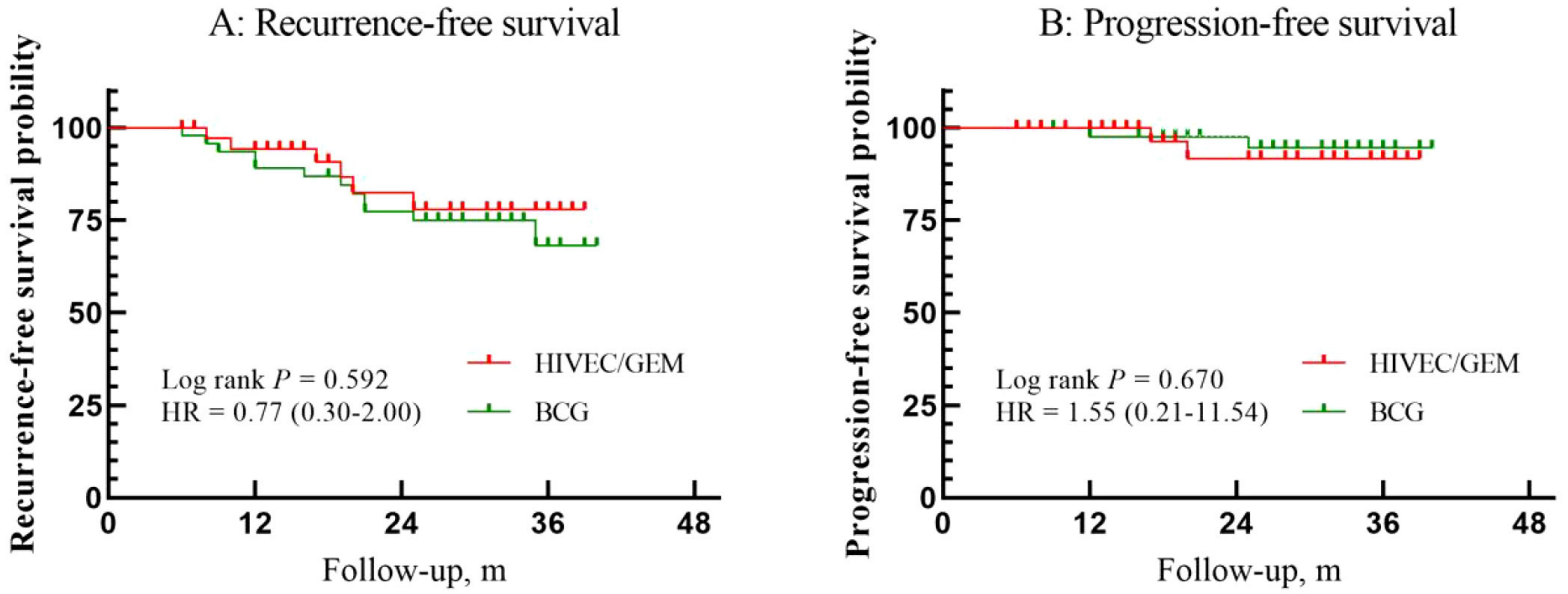
Survival outcomes. Kaplan-Meier curves in patients treated with HIVEC/GEM versus BCG. Red color means HIVEC/GEM, green color means BCG. **(A)** RFS (log rank p=0.592). **(B)** PFS (log rank p=0.670). Vertical lines indicate censored patients.

During the follow-up, 18 patients experienced tumor recurrence (6 in the HIVEC/GEM group and 12 in the BCG group); the recurrence rate was comparable between the two groups (15.8% (6/38) versus 25.5% (12/47), *p* = 0.274). 4 patients (2 in the HIVEC/GEM group, 2 in the BCG group) had disease progression. The 24-month RFS was 78.0% in the HIVEC/GEM group and 75.0% in the BCG group (p = 0.592) (**Figure 1**). The mean time to recurrence was 16.5 ± 6.4 months for the HIVEC/GEM group and 17 ± 8.3 months for the BCG group. The 24-month PFS was 91.7% for HIVEC/GEM and 94.6% for BCG (*p* = 0.670). At 24-month, CSS was 100% in both treatment groups. Among 4 patients with disease progression, 3 patients underwent radical cystectomy, and the other 1 chose bladder-sparing treatment.

Compared with the BCG group, HIVEC/GEM had a significantly lower AEs rate (47.37% (18/38) versus 70.20% (33/47), *p* = 0.033) (**Table 2**). 42.1% (16/38) of patients in the HIVEC/GEM group reported at least one grade 1–2 AE, which is also fewer than in the BCG group (63.8% (30/47)) (*p* = 0.046). Chemical cystitis and pain were the most common AEs in both groups. Most complications were mild (88.9% for the HIVEC/GEM group and 90.9% for the BCG group were Clavien-Dindo grade ≤2) (**Table 2**), and the distribution of severity did not differ between groups. 5 patients (9.4%) reported AEs of grade 3-5. During the treatment process, only one patient who received BCG treatment experienced a grade 4 complication at the 3rd month and subsequently withdrew from the treatment plan.

**Table 2:** Adverse events according to the common terminology criteria for adverse events.

Adverse events, n(%)	Grade 1-2		Grade 3		Grade 4-5	
	HIVEC/GEM	BCG	HIVEC/GEM	BCG	HIVEC/GEM	BCG
Cystitis (frequency/urgency/spasms)	9(23.7)	14(29.8)	1(2.6)			
Pain	7(18.4)	13(27.7)		1(2.1)		
Hematuria	2(5.0)	4(8.5)				
UTI	3(7.9)	4(8.5)	1(2.6)			1(2.1)
Fatigue		7(14.9)				
Other	2(5.0)			1(2.1)		
Any	16(42.1)	30(63.8)	2(5.0)	2(4.3)		1(2.1)

HIVEC, hyperthermic intravesical chemotherapy; GEM, gemcitabine; BCG, Bacillus Calmette–Guerin; UTI, urinary tract infection.

On stepwise multivariate logistic regression analysis (**Table 3**), AEs was positively predicted by Age (OR 1.074, 95% CI 1.004-1.148, p=0.037), HIVEC/BCG (OR 0.238, 95% CI 0.066-0.862, p=0.029), and risk group (OR 0.068, 95% CI 0.018-0.261, p<0.001).

**Table 3:** Multivariable logistic regression model for predictors of adverse events.

Predictor	B	OR(95% CI)	P value
Age	0.071	1.074(1.004-1.148)	0.037
HIVEC/BCG	-1.437	0.238(0.066-0.862)	0.029
intermediate-/high-risk NMIBC	-2.686	0.068(0.018-0.261)	<0.001

HIVEC, hyperthermic intravesical chemotherapy; BCG, Bacillus Calmette–Guerin; NMIBC, non-muscle-invasive bladder cancer.

## Discussion

The additional benefit of HIVEC has been established in some reports, including several prospective randomized clinical trials (3, 6, 15). However, most current research uses HIVEC combined with mitomycin C, which is not commonly used in Asia. In the present study, we compared HIVEC with GEM versus intravesical BCG therapy in patients with intermediate- and high-risk papillary NMIBC. The results suggest that HIVEC with GEM was comparable to BCG in terms of oncological efficacy, with a better performance on treatment safety.

For patients with intermediate- and high-risk NMIBC, further adjuvant intravesical therapy is mandatory because of the considerable likelihood of recurrence and/or progression (1, 2). BCG has been confirmed to be superior to chemotherapy in this situation. RCTs comparing BCG with MMC or epirubicin demonstrated BCG significantly magnified the reduction of recurrence and progression rates in those patients (16, 17). However, despite presumed complete transurethral resection and adjuvant therapy including intravesical BCG instillation, recurrence and progression in NMIBC are still high. Meanwhile, the efficacy of BCG was hampered by drug availability and toxic effects (4). It has been reported that approximately 12% of patients cannot complete maintenance treatment due to local and systemic side effects (18). Therefore, new treatment options are anticipated.

Hyperthermic chemotherapy was firstly approved for treatment of peritoneal metastases and has been investigated in clinical use for NMIBC patients for a long time (4). Urologists want an off-the-shelf alternative to BCG with comparable efficacy and safety. HIVEC significantly improved the efficacy of intravesical chemotherapy. Meta-analysis demonstrated a 59% relative reduction in recurrence of NMIBC when chemohyperthermia was used compared to MMC alone (19). Meanwhile, Plata (20) confirmed in a large-scale population study that HIVEC through MMC is well tolerated for patients with intermediate- and high-risk NMIBC.

The latest researches and a systematic review study have confirmed Intravesical CHT had equivalent oncological outcomes and similar safety profile when compared to BCG maintenance therapy for patients with intermediate- and high-risk NMIBC (21–23). Arends (3) found that heated MMC was superior to BCG on 24-month RFS in intermediate- and high-risk NMIBC patients with papillary tumors (81.8% vs. 64.8%, *p* = 0.02). Guerrero-Ramos (15) also found in the high-risk bladder tumor population, the HIVEC group had a better PFS compared to the BCG group. But recently, two multicenter RCTs showed different results from previous reports (24, 25). They found no additional oncological benefit of HIVEC in intermediate-risk NMIBC compared to MMC alone. This mismatch may be attributed to the differences in patients’ selection, device system, and treatment schedule. Most recent HIVEC research using MMC as an anti-tumor agent shows conflicting results, indicating that further study is needed.

Intravesical instillation of gemcitabine was an alternative choice in the setting of the worldwide shortage of BCG, but encouraging results have aroused the interest of urologists concerning the efficacy and safety. RCT has shown it was well tolerated, with no significant difference in AE profiles compared with intravesical saline instillation (7). Gemcitabine can reduce the recurrence and progress rate in a population of recurrent NMIBC along with fewer CTCAE grade I or II adverse events compared to MMC (26). Researches also investigated the oncological efficacy of GEM versus BCG at room temperature; data shows there were no significant differences in the recurrence and progression rates in the intermediate-risk groups, but GEM was inferior to BCG in the high-risk NMIBC patients (9). Data on hyperthermia combined with GEM was scarce. In our study, HIVEC/GEM demonstrated at least comparable oncological efficacy in terms of RFS and PFS at 24 months in a per-protocol population.

Adverse effects were another thing we were concerned about, since it was critical for ensuring patients’ safety and adherence. AEs in BCG treatment are more common than with intravesical chemotherapy, while GEM is well-tolerated due to its high mucosal absorption and relatively low plasma absorption (18). Meta-analysis shows local side effects occur in 7-40% of patients, while systemic side effects influence less than 10% (18). GEM alone or combined with docetaxel was also used in BCG-unresponsive disease with similar side effect profiles (27). In our study, HIVEC/GEM and BCG have similar AE profiles, but HIVEC/GEM has a significantly lower AE rate compared with the BGC group. The most common AEs of both intravesical therapies were chemical cystitis, pain, and hematuria. Most of AEs were CTCAE grade I or II and reversed soon after treatment. AEs with grade >3 were scarce in both groups and without significant difference. Our data indicates that the combination of GEM with HIVEC doesn’t improve the AE rate vs GEM alone.

To our knowledge, this is the first paper that investigates the efficacy and safety of HIVEC/GEM in an intermediate- and high-risk per-protocol NMIBC population. We found HIVEC/GEM has comparable oncological efficacy and fewer AEs compared with BCG. Given the persistent global BCG shortage, our results highlight HIVEC/GEM as a potential alternative therapy for intermediate- to high-risk papillary NMIBC, which warrants prospective validation.

However, several limitations must be acknowledged. Firstly, the intrinsic nature of retrospective analysis brings unavoidable selection bias, even we prospectively collected the data. Besides, patients with CIS disease or treatment discontinuation due to toxicity were excluded from the present study, which may attenuate the credibility of the results. What’s more, the limited sample size, low number of events, and relatively short follow-up period may have introduced confounding effects on the results. Finally, the conclusions of this study cannot be considered definitive and must be confirmed in larger prospective trials.

## Conclusion

In our retrospective study, we found that HIVEC may have similar oncological efficacy and better safety compared to BCG in patients with intermediate- and high-risk papillary NMIBC. However, this result needs further validation in comprehensive prospective studies.

## Data Availability

The original contributions presented in the study are included in the article/supplementary material. Further inquiries can be directed to the corresponding author.
